# Prevalence of Bleeding Symptoms among Adolescents and Young Adults in the Capital City of Saudi Arabia

**DOI:** 10.1155/2018/1858241

**Published:** 2018-05-02

**Authors:** Tarek Owaidah, Mahasen Saleh, Hazzah Alzahrani, Mahmood Abu-Riash, Ali Al Zahrani, Mohammed Almadani, Ayman Alsulaiman, Abdulmajeed Albanyan, Khawar Siddiqui, Khalid Al Saleh, Abdulkareem Al Momen

**Affiliations:** ^1^Department of Pathology and Laboratory Medicine, King Faisal Specialist Hospital and Research Centre, Riyadh, Saudi Arabia; ^2^Center of Excellence in Thrombosis and Hemostasis, King Saud University, Riyadh, Saudi Arabia; ^3^Pediatric Hematology, King Faisal Specialist Hospital and Research Centre, Riyadh, Saudi Arabia; ^4^Oncology Center, King Faisal Specialist Hospital and Research Centre, Riyadh, Saudi Arabia; ^5^Research Center, King Faisal Specialist Hospital and Research Centre, Riyadh, Saudi Arabia; ^6^Ministry of Education, Riyadh, Saudi Arabia

## Abstract

**Background:**

Bleeding disorders vary in prevalence. While some are rare, some can be common in both sexes. Most bleeding disorders manifest as chronic bleeding tendencies or as an increase in bleeding during surgical procedures or trauma. The consequences of bleeding can be as simple as iron deficiency or catastrophic, resulting in severe morbidity and mortality. Bleeding disorders typically affect both sexes except hemophilia A and B, which mainly affects males.

**Method:**

We conducted a questionnaire-based survey among adolescents and young adults (1901 [49%] boys, 1980 [51%] girls) in Riyadh city regarding bleeding symptoms. Of these, 1849 (47.6%) responded “Yes/Positive” for at least one question about the bleeding symptoms.

**Results:**

The most common bleeding symptom was epistaxis (19.7% of the sample population) detected in Phase I of the study. A tandem survey was conducted among 525 adolescents who had responded “Yes/Positive” to any one of the questions inquiring about bleeding symptoms.

**Conclusion:**

In this study, we report for the first time the prevalence of bleeding symptoms in a representative sample of Saudi adolescents and young adults.

## 1. Introduction

Bleeding disorders are a group of inherited disorders with different prevalence rates depending on many ethnicities. The most known inherited bleeding disorders are hemophilia A and B, which are relatively rare. Hemophilia A affects 1 : 5000–10,000 males, while hemophilia B affects 1 : 50,000–100,000 males. Hemophilia A and B can be very serious and life-threatening for individuals as well as a costly disease for families and countries [1]. von Willebrand disease (VWD) is another bleeding disorder, which is an inherited disorder that is caused by deficiency or dysfunction of VWF. VWD is a relatively common cause of bleeding, but the prevalence varies considerably among studies and depends strongly on the case definition that is used. The prevalence of VWD has been estimated in several countries on the basis of the number of symptomatic patients seen at hemostasis centers and ranges from about 23 to 110 per million population (0.0023–0.01%) [2]. It is also been estimated by screening populations for bleeding symptoms (population-based approach), with estimates reported at 0.6%, 0.8%, and 1.3% [3–7]. These international estimates of the prevalence of VWD do not address ethnicity or geographic variables as potential independent factors, though ethnic variation in VWF levels can influence the diagnosis of VWD [8–10]. Moreover, most mild bleeding disorders are often unrecognized, as patients bleed only during stress periods or with surgery and medical procedures [11, 12]. The most common result of these chronic bleeds is iron deficiency anemia, which is more common in women due to excessive menstrual bleeding.

The few studies estimating the prevalence of VWD by screening populations using formal standardized criteria reported a prevalence approaching 1%, with no ethnic differences [4, 6]. Platelet disorders are another group of bleeding disorders, in which bleeding can result from a decrease in platelet count (thrombocytopenia), with a reported incidence of 1.9 and 6.4 per 10 children/year. In adults, the incidence of idiopathic thrombocytopenic purpura is 3.3 per 10 adults/year [13, 14]. There are additional inherited platelet disorders with a generally unknown prevalence. Inherited thrombocytopathies are a heterogeneous group of platelet disorders present mainly with mucocutaneous bleeding of variable severity caused by defects in platelet adhesion, aggregation, granules, and signal transduction [15]. The diagnosis of more prevalent mild forms of inherited thrombocytopathies is difficult, even with extensive laboratory testing [16]. This could be due to the presence of a very broad range of candidate platelet proteins potentially implicated in the pathogenesis of nonsevere inherited thrombocytopathies, many of which are incompletely characterized [17]. Gresele et al. [18] estimated that only 40–60% of mild platelet disorders can be diagnosed at the level of the defective platelet pathway.

In the Kingdom of Saudi Arabia, no population-based screening studies have examined the prevalence of bleeding disorders, although several case reports and case-series have been published [19–23]. This is potentially important, as Arab populations may have a higher prevalence of bleeding disorders than in the West, primarily owing to the increased rate of consanguinity in Arab communities. The purpose of the current study was to conduct the first screening survey in the capital city of Riyadh focused on the prevalence of bleeding symptoms among adolescents and young adults.

## 2. Material and Methods

We conducted an epidemiological survey on a randomly selected Saudi national adolescent sample of intermediate and high school participants of both sexes in Riyadh using a semistructured validated condensed (MCMDM-1) VWD Bleeding Questionnaire. This questionnaire was selected owing to its capacity to generate quantifiable data from the entire study group [24]. Process of translation into Arabic and adaptation of MCMDM-1 through an expert committee for implementation has been published elsewhere [25]. A shorter questionnaire, derived from the same primary questions but with less detail, was extracted to be used as a primary screening tool for the initial phase of the study, whereas the original questionnaire was used in the second phase of the study only when participants gave a positive response to any primary question. The survey was conducted onsite by trained Arabic speaking interviewers. All questionnaires were coded for data entry. The process involved the following phases.

*Phase I.* Fifty schools (30 intermediate and 20 secondary) were randomly selected from a complete list of intermediate and secondary schools in Riyadh city (Table 1). We distributed invitations to the schools with the intention to have at least 100 participants from each school. An initial visit was paid to participating schools to explain the aim of the study and to distribute educational materials on bleeding disorders. Interviews were conducted after obtaining signed assents or consent, depending upon the age of the participant. The Phase I data were analyzed to identify participants who gave a positive response to any of the primary questions; these participants were considered to potentially have a bleeding tendency.

*Phase II.* Respondents with at least one positive response were contacted again for getting further details regarding symptoms and to assess potential recall bias. Their responses were recorded using a detailed questionnaire further probing on the bleeding type specific to the site of bleeding, based upon MCMDM-1.

### 2.1. Data Management and Quality Assurance

Arabic speaking trained individuals interviewed participants and collected data using specially designed Arabic-language Case Report Forms (CRF). Confidentiality was maintained by assigning each participant a unique identification number, which was entered into a computerized database. Data were validated for data entry errors by cross checking the improbable answers. Discrepancies were handled by reviewing the original forms. All data were transferred to IBM SPSS Statistics Version 20 (IBM Corp., Armonk, NY, USA) for final analysis.

Processed data are reported in percentages along with the denominator which defines the available data. To compare categorical data, Chi-Square or Fisher's exact test was used while Shapiro-Wilk test was utilized to test for the normality of continuous data. *P* value of less than 0.05 was considered as achieving statistical significance.

## 3. Results

### 3.1. Phase I

During Phase I, 3923 randomly selected students were approached and told about the study after providing written literature and assent forms with an invitation to be interviewed regarding bleeding tendency. Of these 98.9% (3881, male: 1901 [49%]; female: 1980 [51%]) gave assent to participate. Forty-two (42) refused to participate. Median age of the participants at the time of interview for available data was 18.2 years (*n* = 3322; range, 12.0–21.0 years; mean ± standard deviation: 17.8 ± 1.4 years; *P* value for normality < 0.001). There were 40 (1.2%) participants < 14 years, 1340 (40.3%) 14–17 years, and 1942 (58.5%) ≥ 18 years. Of 3881 participants who completed the survey, 1849 (47.6%) answered “yes/positive” to at least one of the eight questions (Table 2).

Of 72 (1.9%) participants who responded “Yes” to “Have you ever been diagnosed with any bleeding disorder?” question, eight reported having hemophilia, three platelets disorders, and none reported VWD; remaining participants did not disclose additional information. In response to a family history of any bleeding disorders, 237 out of 3730 (6.4%) participants who opted to answer, responded positively, 44 hemophilia, 22 platelet disorders, and 3 VWD; the remaining 167 did not provide any details or did not know the exact disorder.

### 3.2. Phase II

**Figure 1:**
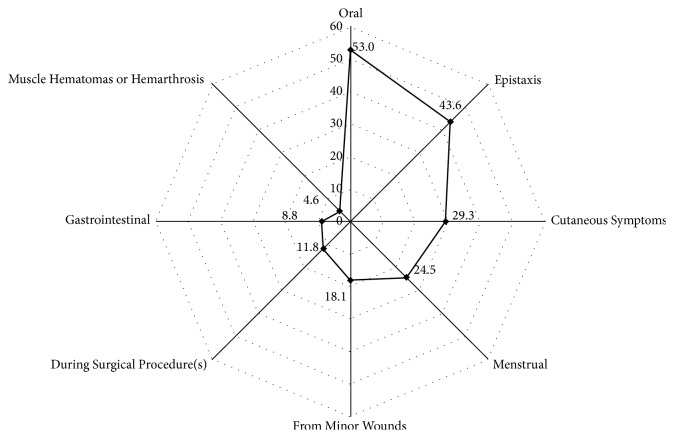
Prevalence of bleeding symptoms as reported by the participants.

Of the 1849 (47.6%) participants who responded “yes” to at least one question in Phase I, only 525 (28.4%; male: 296 [56.4%]; female: 229 [43.6%]) replied to our call to participate in Phase II of the study and attend the second interview. Reasons for those participants of Phase I who had possibly exhibited bleeding symptoms during the interview, not completing the Phase II of the study, included wrong or changed phone numbers, participant moving out of the area, or incorrect contact details. Median age at the time of interview for this group of participants was 18.4 years (range: 12.5–20.9 years; mean  ±  standard deviation: 18.1 ± 1.4; *P* value for normality < 0.001). There were eight (1.8%) participants < 14 years, 140 (31.8%) 14–17 years, and 292 (66.4%) ≥ 18 years of age. Data on age at interview was available for 440 participants. A total of 442 participants of the Phase II of the study answered positive to any of the questions inquiring about the symptoms pertaining to the bleeding disorders (Table 3); thus an overall prevalence of all bleeding symptoms was 84.2% (Figure 1).

### 3.3. Oral Cavity Bleeding

About fifty-three percent (278/525, 52.9%) of participants reported oral cavity bleeding. Of these, 85.3% (237/278) reported bleeding from the mouth while brushing their teeth, 20.5% (57/278) spontaneously from the gums, 11.2% (31/278) from lip or tongue bites, and 1.8% (5/278) from tooth eruption. In addition, 7.2% (20/278) reported having sought medical attention, 75% (15/20) sought consultation only, while one participant reported having undergone a blood transfusion. Oral cavity bleeding was significantly higher in girls (134/229, 58.5%) than in boys (144/296, 48.6%, *P* = 0.028). Similarly, oral bleeding from lip or tongue bites was significantly higher in girls (25/134, 18.7%) than in boys (6/144, 4.2%, *P* < 0.001).

### 3.4. Epistaxis

The next most common symptom was epistaxis, which was reported by 229 (43.6%) participants and was more predominant among boys (147/296, 49.7%) than girls (82/229, 35.8%, *P* = 0.002). Epistaxis mostly occurred 1–5 times yearly (118/228, 51.8%), lasted 1–10 minutes (111/223, 49.8%), and was spontaneous in 87.2% (197/226) of participants. It primarily occurred at a single nostril (149/225, 66.2%) and was not related to ingestion of any drugs (227/228, 99.6%), though 56.2% (127/226) reported a seasonal relationship. A majority (162/221, 73.3%) reported successful cessation of epistaxis with short compression, while some (57/221, 25.8%) reported spontaneous cessation, and a few (2/221, 0.9%) reported cessation following medical intervention. No significant dependence was found between the age of maximum severity (<14 years, 111/219, 50.7%; ≥14 years, 108/219, 49.3%). A small proportion of participants (36/227, 15.9%) reported that they had sought medical attention in the past due to epistaxis, including consultation only (66.7%  [24/36]), cauterization (25.0%  [9/36]), and packing (2.8%  [1/36]). No participants reported needing blood transfusion.

There was a significant difference in the spontaneity of epistaxis between sexes (girls: 77.81 [95.1%]; boys: 120/145 [82.8%], *P* = 0.007). Moreover, 40.6% (58/143) of boys reported having experienced bleeding from both nostrils compared to 22% (18/82) of girls (*P* = 0.005).

### 3.5. Cutaneous Symptoms

Cutaneous symptoms were reported by 29.3% (154/525), most commonly occurring six times yearly (17/92, 18.5%) and ranging from one time (8/92, 8.7%) to 24 times (3/92, 3.2%). The most common manifestation was bruises (109/142, 76.8%), followed by hematoma (16/142, 11.3%); these were most commonly manifested at exposed sites (97/139, 69.8%). Only 14.2% (18/127) sought medical attention regarding this, nine of whom sought consultation only. Cutaneous symptoms occurred more commonly in girls (102/229, 44.5%) than in boys (52/296, 17.6%, *P* < 0.001). In addition, more boys (12/45, 26.7%) required medical attention than girls (6/82, 4.7%, *P* = 0.006).

### 3.6. Menstrual Bleeding

Heavy menstrual bleeding was reported by 24.5% (56/229) of female participants. Median duration of menstruation was 7 days (range: 3–15 days; mean: 6.5 days; *P* for normality < 0.001), and the median number of heavy days was 3 (range: 0–8; mean = 2.7, *P* for normality < 0.001). The most commonly reported duration of menstruation was 7 days (40.9%  [90/220]), and the most commonly reported number of heavy days was 3 (36.9%  [73/198]). Medical attention was sought by 21.4% (12/56); three had consultation only, and nine received iron supplements.

### 3.7. Minor Wound Bleeding

Bleeding from minor wounds was reported by 18.1% of participants (95/525), with 52.6% (41/78) reporting this occurred 6–12 times a year, and 25.6% (20/78) reporting >12 times a year. Average duration of a single episode was 1–10 minutes in 78.3% (65/83), > 10 minutes in 19.3% (16/83), and <1 minute in only 2.4% (2/83). Location of minor wounds was primarily exposed sites (89.6%, 69/77), and there was minimal or no trauma in 67.4% (64/95). Only 8.4% (8/95) needed medical attention, with two participants needing surgical hemostasis. Bleeding from minor wounds was more common in girls (56/229, 24.5%) than in boys (39/296, 13.2%, *P* = 0.001).

### 3.8. Bleeding during Surgery/Tooth Extraction

Those who underwent at least one surgery of any type comprised 30.5% (160/525). Among these, 112 (70%) had one surgical episode, 33 (20.6%) had two, seven (4.4%) had three, three (1.9%) had four, four (2.5%) had five, and one (0.6%) had seven, thus totaling 238 reported surgical episodes. Among those who underwent any surgery, 24 (15%) reported at least one surgery followed by a bleeding episode and four (2.5%) reported two surgeries followed by bleeding episodes; thus 28/238 reported surgeries with a postsurgery bleeding episode alluding to a prevalence of 11.8%. Bleeding episode after first surgery was reported by almost one-quarter of the participants (39/160, 24.4%) and was significantly higher in girls (19/52, 36.5%) than in boys (20/108, 18.5%, *P* = 0.018). Types of surgeries included major abdominal surgery (4/160, 2.5%), major thoracic surgery (2/160, 1.2%), and molar extraction or dental surgery (28/160, 17.5%). Posttooth extraction bleeding was reported by 12.8% (67/525) of participants and was significantly higher in girls (42/229, 18.3%) than in boys (25/296, 8.4%, *P* = 0.001). No action was required to control the bleeding in 47 participants, resuturing was performed in nine, and only one required blood transfusion.

### 3.9. Gastrointestinal Bleeding

GI bleeding was reported by 8.8% (46/525) of participants. Six (6/16, 37.5%) reported having had GI bleeding at least once, four (25%) at least three times, two (12.5%) at least five times, two (12.5%) at least 10 times, one (6.2%) at least two times, and one (6.2%) at least 12 times. Of these, 39.1% (18/46) had hematemesis, 37% (17/46) had hematochezia, and 13% (6/46) had melena. In addition, 23.8% (10/42) had associated GI disease; of these, 30% (3/10) reported ulcer and 10% (1/10) angiodysplasia, while none reported portal hypertension. Moreover, 28.3% (13/46) mentioned that they sought medical attention for this, 84.6% (11/13) of whom had consultation only, while one female participant (7.7%) underwent a blood transfusion. GI bleeding was more common in girls (31/229, 13.5%) than in boys (15/296, 5.1%, *P* = 0.001).

### 3.10. Muscle Hematoma and Hemarthrosis

Muscle hematomas or hemarthrosis was reported by 4.6% (24/525), with spontaneous bleeding in 37.5% (9/24), which was higher in girls (6/10, 60%) than in boys (3/14, 21.4%, *P* = 0.092). Two of these participants (2/24, 8.3%) reported that they sought medical attention, one was given replacement therapy, and complete data was not available for the other. None received desmopressin or blood transfusion.

### 3.11. Other Types of Bleeding

A total of twelve (12/525, 2.3%) participants reported experiencing episodes of bleeding other than the above-mentioned types. Only two of these (2/12, 16.7%) went to see a medical practitioner; one was given replacement therapy and the other received consultation only. No data was available regarding the type of bleeding.

More than half (53.1%, *n* = 279) of the students (525) reported bleeding episodes from more than one group of sites. Majority of these (53%, *n* = 148) were females (*P* value: 0.001). Oral cavity bleeding with cutaneous symptoms was the most common (12.2%, 18 out of 148), followed by oral cavity bleeding with epistaxis (6.8%, 10 out of 148) and oral cavity bleeding with epistaxis and cutaneous symptoms (4.7%, 7 out of 148). Among boys reporting bleeding episodes observed from multiple sites (*n* = 131), oral cavity bleeding with epistaxis (31.3%, 41 out of 131) was the most frequently observed kind, followed by oral cavity bleeding with cutaneous symptoms (7.6%, 10 out of 131) and epistaxis with cutaneous symptoms (5.3%, 7 out of 131).

## 4. Discussion

To our knowledge, this is the first largest screening study attempting to address the estimation of prevalence of symptoms of bleeding disorders in the capital city of Saudi Arabia. Prior studies from Saudi Arabia reporting the same have been smaller, hospital-based studies. For instance, El-Bostany et al. [19] assessed the local prevalence of some inherited bleeding disorders in pediatric patients which involved 43 children with various bleeding manifestations recruited from a children's hospital in Cairo, Egypt, and Jeddah, Kingdom of Saudi Arabia. Of these, 12 (27.9%) had VWD, 11 (25.5%) had hemophilia A, three (7%) had hemophilia B, seven (16.3%) had platelet disorders, and 10 (23.3%) had bleeding of undiagnosed cause. In addition, Ahmed et al. [20] reported 34 cases of inherited bleeding disorders from Eastern Province of Saudi Arabia; of these, 15 had hemophilia, one had factor VII deficiency, one had factor X deficiency, 12 had Glanzmann thrombasthenia, and five had unidentified platelet function disorders. Moreover, Al-Sharif et al. [21] reported clinical phenotype of around 20 patients with factor XIII deficiency in the Riyadh region. Furthermore, Al-Fawaz et al. [22] conducted an 8-year retrospective analysis of patients referred for suspected inherited bleeding disorders in the Riyadh region and found 168 patients had bleeding symptoms that fulfilled the criteria for inherited bleeding disorders. Of these, 41 (24.4%) had hemophilia A, 16 (9.5%) had hemophilia B, 25 (14.9%) had VWD, 18 (10.7%) had Glanzmann thrombasthenia, 18 with Bernard-Soulier disease, five (3.0%) had factor XI deficiency, two (1.2%) had factor XII deficiency, four (2.4%) had factor V deficiency, four (2.4%) had factor VIII deficiency, one (0.6%) had factor VII deficiency, two (1.2%) had dysfibrinogenemia, and one (0.6%) had afibrinogenemia. Additionally, Islam and Quadri [23] conducted a 7-year retrospective review of all hospitals in Eastern Province of Saudi Arabia. They reported 54 patients diagnosed with hereditary coagulation factor deficiencies, including 42 hemophiliacs, 5 with probable factor XIII deficiency, and 7 with VWD. There are also rare reports from other Arab countries reporting small hospital-based studies [26–28].

In the current study, boys experienced epistaxis more frequently, which was more likely to be spontaneous, to occur at both nostrils, and to have seasonal differences; this may be explained by the more outdoor lifestyle in boys in a dry, hot environment, which leads to more nasal dryness and is one of the common causes of epistaxis in general [29–31]. In contrast, girls wear veils and are usually covered in the outdoor setting, which may reduce nasal dryness.

A study from Sweden among healthy university females showed a high prevalence of bleeding symptoms including menorrhagia. 73% of the participants had one bleeding symptom while 43% had more than one symptom [32]. Another study from Turkey done on female university residents showed 82/376 (22%) healthy females reporting menorrhagia, after excluding pelvic pathology out of 11/76 (14.5%) were found to have an underlying bleeding disorder [33].

This study provides insight into the existence of various bleeding disorders and highlights the need for a national surveillance system for identifying the individuals in the early age with such disorders. There is a need for genetic mapping of families suffering from bleeding diathesis in order to prevent further generations of Saudi nationals being affected. Specialized hematological investigations from a nationally representative sample would provide more insight into the nature and classification of the more prevalent disorders and guide treatment and prevention. Although every citizen has an easy access to the healthcare services in the Kingdom of Saudi Arabia, it is imperative to improve the quality of life of the affected individuals and families by raising awareness and reducing exposure to precipitating insults. Genetic counseling of the severely affected families is mandatory for genetically transmitted disorders.

It might be said that the participants in this sample were more aware of their health problems since they were residing in an urban area; the importance to search for the prevalence in other suburban and rural areas of the country is also highlighted, since consanguinity maybe more prevalent in those closed populations. Generally it was observed that girls reported higher prevalence of bleeding symptoms than boys. We believe this could be a reporting bias since girls are generally more self-caring than boys especially in the teenage years. Boys tend to ignore minor cuts and bruises and may attribute them to their typical physical activities.

## 5. Limitations

This report is limited by the lack of laboratory related data further identifying specific bleeding disorders. It was beyond the feasibility of the research with respect to logistics and financial support. It would be very interesting to observe the prevalent forms of bleeding disorders in a future report hence guiding the policy makers for efficient resource allocation.

## 6. Conclusion

This survey which is the first epidemiological study for bleeding symptoms in Saudi Arabia using standardized tool (MCMDM-1) that had highlighted the need to conduct a national survey in the Kingdom on broader representative sample with extensive laboratory test to explore the prevalence of different bleeding disorders. We also recommend that physicians be cautious of the existence of bleeding disorders in the community as minor symptoms can get easily ignored and lead to a catastrophe when challenged by trauma or surgery. Also, a sustainable public awareness program focusing the early diagnosis, treatment, and genetic counseling among the residents of the regions with high prevalence of bleeding disorders should be initiated.

## Figures and Tables

**Table 1:** Sampling frame for determining the prevalence of bleeding symptoms.

	Intermediate schools			High schools		
	Number of participants	Number of schools	Number of clusters	Number of participants	Number of schools	Number of clusters
Boys	81,869	336	15	72,621	194	10
Girls	84,607	419	15	80,136	293	10
Total	166,476	755	30	152,757	487	20

**Table 2:** Responses to Phase I of the Survey (*n* = 3881).

Questions	Male *n* (total), %	Female *n* (total), %	Total *n* (total), %	*P* value
(1) Previous diagnosis of any bleeding disorder?				
Yes	42 (1901), 2.2	30 (1980), 1.5	72 (3881), 1.9	0.069
Did not reply	-	-	-	
(2) Previous episodes of epistasis?				
Yes	453 (1901), 23.8	311 (1980), 15.7	764 (3881), 19.7	<0.001
Did not reply	-	-	-	
(3) Bleeding under the skin?				
Yes	218 (1840), 11.8	406 (1931), 21.0	624 (3771), 16.5	<0.001
Did not reply	61	49	110	
(4) Postsurgery bleeding?				
Yes	97 (1834), 5.3	313 (1930), 16.2	410 (3764), 10.9	<0.001
Did not reply	*67*	*50*	*117*	
(5) Bleeding from the mouth?				
Yes	161 (1801), 8.9	188 (1928), 9.8	349 (3729), 9.4	0.399
Did not reply	*100*	*52*	*152*	
(6) Bleeding from the digestive system?				
Yes	107 (1834), 5.8	211 (1930), 10.9	318 (3764), 8.4	<0.001
Did not reply	*67*	*50*	*117*	
(7) Postdental extraction bleeding?				
Yes	314 (1823), 17.2	126 (1926), 6.5%	440 (3749), 11.7	<0.001
Did Not Reply	*78*	*54*	*132*	
(8) Muscular bleeding?				
Yes	94 (1725), 5.4	201 (1894), 10.6	295 (3619), 8.2	<0.001
Did Not Reply	*176*	*86*	*262*	
(9) Family history of bleeding disorders?				
Yes	146 (1821), 8.0	91 (1909), 4.8	237 (3730), 6.4	<0.001
Did Not Reply	*80*	*71*	*151*	
(10) Any other bleeding disorders? (for boys only)				
Yes	173 (1882), 9.2	-	173 (1882), 9.2	-
Did Not Reply	*19*	*-*	*19*	
(11) Heavy menstrual bleeding (girls only)				
Yes	-	330 (1980), 16.7	330 (1980), 16.7	-
Did Not Reply	-	*-*	*-*	
(12) Any Question 2 through 8 or 10				
Yes	832 (1901), 43.8	1017 (1980), 51.4	1849 (3881), 47.6	<0.001
Did Not Reply	-	-	-	

Responses left blank or *Did Not Reply *are not included in the final calculations.

**Table 3:** Responses to Phase II of the Survey (*n* = 525).

Symptom	Male *n* (total), %	Female *n* (total), %	Total *n* (total), %	*P* value
Oral cavity bleeding	144 (296), 48.6	134 (229), 58.5	278 (525), 52.9	0.028
Epistaxis	147 (296), 49.7	82 (229), 35.8	229 (525), 43.6	0.002
Cutaneous symptoms	52 (296), 17.6	102 (229), 44.5	154 (525), 29.3	<0.001
Menstrual bleeding	Not applicable	56 (229), 24.5	56 (229), 24.5	-
Minor wound bleeding	39 (296), 13.2	56 (229), 24.5	95 (525), 18.1	0.001
Bleeding during tooth extraction	25 (296), 8.4	42 (229), 18.3	67 (525), 12.8	0.001
Gastrointestinal bleeding	15 (296), 5.1	31 (229), 13.5	46 (525), 8.8	0.001
Muscle hematoma and hemarthrosis			24 (525), 4.6	
Spontaneous bleeding	3 (14), 21.4	6 (10), 60	9 (24), 37.5	0.092

Values are reported as *n* (N), %.
